# Exploring when and how adolescents sit: cross-sectional analysis of activPAL-measured patterns of daily sitting time, bouts and breaks

**DOI:** 10.1186/s12889-019-6960-5

**Published:** 2019-06-11

**Authors:** Lauren Arundell, Jo Salmon, Harriet Koorts, Ana Maria Contardo Ayala, Anna Timperio

**Affiliations:** Geelong, Australia, Institute for Physical Activity and Nutrition (IPAN), School of Exercise and Nutrition Sciences, Deakin University, 221 Burwood Highway, Burwood, Victoria Australia

**Keywords:** Sedentary behaviour, Sitting, Adolescents, Sedentary breaks, Bouts, prolonged sitting, School, Sex differences, ActivPAL

## Abstract

**Background:**

This study describes patterns of adolescents’ objectively-measured sitting volume, sitting bouts, and breaks in sitting during different days and periods of the day, and explored differences by sex and weekdays versus weekend days.

**Methods:**

*Activ*PAL™ data were collected in August 2014–December 2015 from adolescents attending secondary government schools in Melbourne Australia. Eight periods (early morning, mid-morning, morning break, late morning, lunch, early afternoon, late-afternoon and evening) were extracted for each day. School time, class time and out-of-school time were also extracted for weekdays. The percentage of time sitting, percentage of each hour in prolonged sitting (sitting bout ≥10 min), and number of sitting breaks/hour were calculated for each period. Differences by sex, and week and weekend days were determined using t-tests.

**Results:**

Participants (*n* = 297, 15.4 ± 1.6 years) spent 68% of their day sitting; ~ 30% of each hour in prolonged sitting and 3.1 sitting breaks/hour. Sitting time was greater during class time (75%) and school (70%) compared to out-of-school time (65%). Sitting patterns differed between week and weekend days for all periods except the evening period. Girls had higher proportion of sitting during class than boys (76% vs 72% respectively) and school hours (72% vs 67%), more prolonged sitting during school hours (27% vs 23%), and more sitting breaks per hour during out-of-school time (2.6 vs 2.4), but fewer during class (2.5 vs 3.3) and school hours (2.7 vs 3.3). Sitting patterns did not differ by sex on weekend days.

**Conclusions:**

Adolescents spent two-thirds of their waking hours sitting, with distinct patterns on weekdays and weekend days. Even though boys and girls were exposed to the same school day routine, girls spent more time sitting and had fewer sitting breaks. Class times, school breaks and the evening period were identified as key intervention periods. Further research is needed to understand the behavioural differences, and guide future intervention design.

**Electronic supplementary material:**

The online version of this article (10.1186/s12889-019-6960-5) contains supplementary material, which is available to authorized users.

## Background

Sedentary behaviour among youth is increasingly being recognised as an independent risk factor for poor physical, social and mental health [1]. Sedentary behaviour is defined as any waking behaviour requiring minimal energy expenditure (≤1.5 metabolic equivalents, METs) and in a sitting, reclining or lying position [2]. A 2016 systematic review showed that among children and youth, elevated sedentary behaviour, specifically screen- based behaviours such as TV viewing and computer use, is positively associated with cardio-metabolic risk factors, unfavourable body composition, low fitness, poor behavioural conduct and low self-esteem [1]. Accordingly, government in countries such as Australia and Canada recommend that children and youth limit their recreational screen time to less than two hours per day [3, 4]. However, only 36% of Australian children (5–11 years) and 21% of adolescents (12–17 years) achieve these public health guidelines [5]. Age-related increases in sedentary behaviour are frequently reported [5–7], placing adolescents at an increased risk of negative health outcomes.

Recent iterations of sedentary behaviour guidelines have also recommended youth reduce and break up long periods of sitting as often as possible [3, 4], as emerging evidence shows that the manner or pattern (i.e. bouts of sitting and breaks in sitting) in which sedentary time is accumulated may also affect health. In lab-based studies, diabetes risk factors (e.g. postprandial glucose and insulin) are reduced when periods of sitting are broken up with light- or moderate intensity activity among overweight adults [8] with moderate-to-vigorous intensity physical activity in healthy weight adolescents [9]. Cross- sectional population studies have also shown that adults who have more breaks in sitting time have lower diabetes and cardio-metabolic indicators, independent of total sitting [10, 11]; however, the relationship between patterns of objectively-measured sedentary time and health outcomes among adolescents is less clear. A recent systematic review and meta-analysis found limited and inconsistent associations between sedentary behaviour patterns of youth and health [12]. This review did also note that most of these studies were based on accelerometer (movement) rather than postural measures (e.g. inclinometer) of sitting.

Accelerometer studies indicate that adolescents’ daily ‘sedentary time’, defined as < 100 counts per minute, ranges from 51 to 74% of the day [13–16], with 25% of the day spent in bouts of 30+ minutes [13] and an average of four breaks in sedentary time per hour [16]. Further, adolescents perform more bouts of sedentary time per hour on weekdays compared to weekend days and fewer bouts of sedentary time during school hours compared to after-school and evening periods [14]. While these studies suggest that sedentary behaviour patterns vary between days and across the day, accelerometer-determined ‘sedentary time’ represents periods of limited movement with posture not being captured.

Studies using monitors to provide postural data and capture sitting (or lying) among adolescents based on the horizontal incline of the thigh are typically small (< 20 participants [17]) or limited only to females [18–20]. These studies indicate that, on average, adolescents spend 52–80% of their day sitting [17, 20], however there may be differences between boys’ and girls’ sitting patterns which is unable to be determined in female only samples. Only one of these studies reported the number of breaks in sitting (54 breaks/day) [17], but did not consider the context (i.e. school time, after-school, during class time) or variations between week and weekend days. Such detail is required for informing targeted intervention development; for example, what periods of the day are prone to, or resistant to prolonged sitting, and whether to target reductions in accumulated and prolonged sitting bouts in the home or school setting, or during class- or school break time. Exploring sitting behaviours and patterns may also be useful for informing future studies that investigate associations with health outcomes and for informing future guidelines. This study aimed to describe patterns of objectively- measured sitting among adolescent boys and girls during different periods of the day on weekdays and weekend days.

## Methods

### Sample

The study used baseline data from the The NEighbourhood Activity in Youth Project (NEArbY study). Level 1 Statistical Areas (SA1) of Melbourne were classified based on median values of area-level income [21] and walkability [22] as high income/high walkability, high income/low walkability, low income/high walkability, and low income/low walkability. Schools (*n* = 137) located across these were invited to participate. Eighteen schools consented (response rate 14%) and students in year levels selected by the school were invited to attend a recruitment presentation at school and take a recruitment pack. In total, 528 returned consent forms. In addition to the core study component (i.e. online adolescent survey), parents could opt-in to their child wearing an *activ*PAL™ monitor (387 opted in) and were also asked to complete a parent survey. Of those that opted to wear an *activ*PAL™, 30 were absent for data collection, leaving a potential sample of *n* = 357 for the current study.

### Ethics

Ethical approval was received from the Deakin University Human Ethics Advisory Group (HEAG-H 152_2013). Approval was also obtained from the Department of Education and Training (2013_002182) and the Catholic Education Office (Project ID #1950). The school Principal provided written consent prior to their students being invited to participate. Written parental consent and participant assent was obtained.

### Measures

Sitting time was assessed using the *activ*PAL™ monitor (PAL Technologies Ltd., Glasgow, Scotland). To produce postural data, accelerometer-derived information about the thigh position is obtained through proprietary algorithms. This function classifies an activity into periods of sitting/lying, standing and stepping, detects transitions between these postures and activities [23] and has acceptable validity in adolescents [24] and children [25, 26]. Participants were asked to wear the *activ*PAL™ during waking hours for eight consecutive days on an elastic garter positioned at the mid-anterior aspect of the right thigh and removed during water-based activities. Participants’ age and sex were reported in an adolescent or parent survey.

### Data management and analysis

The raw *activ*PAL™ data files were analysed in 15-s epochs using specifically-developed excel macros and Stata code (Stata 12). Non-wear time was defined as 60 consecutive minutes of zero counts from the vertical axis of the accelerometer [27]. To be included in analyses for weekdays, participants were required to have worn the *activ*PAL™ for 8 h on at least three weekdays, and to be included in analyses for weekends participants were required to have worn the *activ*PAL™ for 7 h on at least one weekend day [28]. Each weekday was segmented into eight periods according to each schools’ timetable. The periods were *early morning* (6 am to school start time, average length: 2 h, 52 mins), *mid-morning* (start of school to recess start time, average length: 1 h, 51 mins), *morning break* (recess, average length: 25 mins), *late morning* (end of recess to start of lunchtime, average length: 1 h, 41 mins), *lunch* (start to end of lunch, average length: 47 mins), *early afternoon* (end of lunch to end of school, average length: 1 h, 34 mins), *late afternoon* (end of school to 6 pm, average length: 2 h, 53 mins) and *evening* (6 pm to 10 pm, average length, 4 h). To enable comparisons, the weekend days were segmented into the same periods. Three additional periods were calculated for weekdays*: class time* (sum of mid-morning, late morning and early afternoon, average length 5 h, 6 mins); *school time* (sum of mid-morning, morning break, late morning, lunch and early afternoon, average length: 6 h, 18 mins); and *outside of school time* (sum of early morning, late afternoon and evening, average length: 9 h, 45 mins). To be included in the analysis of periods, participants were required to have worn the *activ*PAL™ for at least 50% of the period on at least three weekdays (weekday analysis), and at least 50% of the period on at least one weekend day (weekend analysis) respectively [29, 30].

In total 297 participant provided valid *activ*PAL™ data and 60 did not meet the inclusion criteria. There were no differences in age or sex between participants who did and did not provide any valid *activ*PAL™ data, nor were there any differences in age or sex between participants who only provided valid weekend data and those who provided valid data for both weekdays and weekends.

In line with recommendations from the Sedentary Behaviour Research Network, prolonged sitting was defined as a bout of sitting lasting ten or more minutes [2]. A bout commenced when a complete 15-s epoch occurred in which the identified posture was sitting, and the bout ended when the participant transitioned to an upright posture. A sitting break was defined as the transition from sitting to standing or stepping (using sit-to-stand transition data) [2]. A minimum of 10-s in an upright position were required to register a sitting break in line with the commonly used default settings from the manufacturer [31]. The percentage of a day spent sitting was calculated as the minutes of daily sitting, divided by daily wear time, multiplied by 100. The percentage of each period spent sitting was calculated as the minutes of sitting in the period, divided by wear time in the period, multiplied by 100. The percentage of an hour within each period spent in prolonged sitting was calculated as total minutes of sitting in bouts of ≥10 min divided by the length of the period in hours, multiplied by 100. The frequency of sitting breaks per hour in each period were calculated as the number of sit-to-stand transitions divided by the length of the period in hours.

All outcomes were tested to meet the assumptions of the statistical analysis methods. Paired t-tests were used to determine differences in sitting patterns between weekdays and weekend days. All outcomes were tested for approximate normality as required by paired t-tests [32]. Differences between boys and girls were determined via independent samples t-tests and Welch t-tests when unequal variance occurred, as determined by Levene’s test for equality of variances.

## Results

The final sample with valid weekday data consisted of 297 participants (41% boys) aged 15.4 (±1.6) years. On average, participants, spent significantly more time sitting (Table 1) and had higher wear time on weekdays compared to weekends. Participants spent approximately 30% of each hour in prolonged sitting and performed over three breaks per hour in sitting on weekdays and weekends. The only significant sex difference was observed for prolonged sitting per hour on weekdays, with girls spending more time in prolonged sitting than boys (30.1% vs 27.6%). Sex differences in weekday sitting duration approached significance (*p* = 0.053).

**Table 1:** Patterns of adolescents’ sitting (mean, SD) by sex and day

	All sample (*n* = 297)		Boys (*n* = 119)		Girls (*n* = 178)	
	Weekday^a^	Weekend day^b^	Weekday^a^	Weekend day^b^	Weekday^a^	Weekend day^b^
*Sitting*						
Minutes/day	574.4 (102.68) ^¥^	514.0 (134.24)	560.93 (119.8) ^¥^	530.3 (145.0)	583.8 (88.6) ^¥^	502.7 (125.5)
% of day	68.3 (9.82)	67.9 (13.41)	67.3 (10.56)	69.9 (12.3)	69.1 (9.23)	66.8 (14.0)
*Prolonged sitting*						
% of an hour	28.9 (6.06)	29.7 (8.86)	27.6 (7.03)*	29.6 (8.41)	30.1 (5.08)	29.8 (9.16)
*Sitting breaks*						
Number per hour	3.12 (1.01)	3.15 (1.33)	3.23 (1.17)	3.16 (1.42)	3.18 (0.93)	3.14 (1.28)
*Wear time*	847.55 (93.28) ^¥^	749.95 (123.17)	825.84 (111.71)	760.21 (129.03)	838.56 (90.96)	743.17 (119.32)

^¥^significant differences between weekdays and weekends *p* < 0.05; *significant differences between boys and girls; ^a^ Includes only those with 8 h of wear time on at least 3 weekdays; ^b^ Includes only those with 7 h of wear time on at least one weekend day. This study was conducted in Melbourne, Australia, between August 2014–December 2015

### Proportion of time sitting

**Fig. 1:**
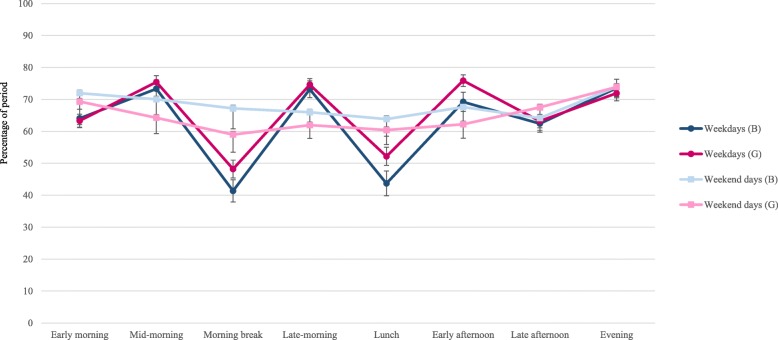
The percentage of each period spent sitting among boys and girls on weekdays and weekend days. Note: B = boys; G = girls. This study was conducted in Melbourne, Australia, between August 2014–December 2015

Table 2 shows that on weekdays, participants spent 43 to 75% of their time within a period sitting, with the highest percentage of sitting occurring during class time and its components (mid-morning, late-morning and early afternoon) and the lowest occurring during morning break and lunch. Besides from the evening period, there were significant differences in sitting time between weekdays and weekend days for all periods. Compared to weekend days, on weekdays adolescents perform higher levels of sitting during mid-morning, late morning and early afternoon periods and lower levels of sitting during morning break and lunch. The greatest difference in sitting between week and weekend days occurred during the morning break (45% vs 62% respectively) and lunch periods (49% vs 62% respectively). Girls spent a greater percentage of the period sitting than boys during the class time (76.1% vs 72.4%) school time (71.5% vs 66.6%), morning break (48.2% vs 41.4%), lunch (52.2% vs 43.7%) and early afternoon (75.9% vs 69.2%) periods (Fig. 1 and Additional file 1: Table S1). There were no sex differences in sitting during any period on weekend days.

**Table 2:** The percentage of time sitting, in prolonged sitting, and the number of sitting breaks in each period

	% of period sitting			% in prolonged sitting (per hour)			Number of sitting breaks (per hour)		
	Weekday^a^	Weekend day^b^	p for day	Weekday^a^	Weekend day^b^	p for day	Weekday^a^	Weekend day^b^	p for day
	Mean %/period (95%CI)	Mean %/period (95%CI)		Mean %/hour (95%CI)	Mean %/hour (95%CI)		Mean #/hr. (95%CI)	Mean #/hr. (95%CI)	
Early morning	63.59 ^¥^ (61.92, 65.26)	70.46 (65.37, 75.54)	< 0.01	18.55 (17.55, 19.51)	19.71 (16.72, 22.70)	0.13	1.75 (1.65, 1.85)	1.62 (1.34, 1.91)	0.08
Mid-morning	74.58 ^¥^ (72.95, 76.21)	66.59 (62.72, 70.20)	< 0.01	23.98 (22.76, 25.19)	25.89 (23.27, 28.51)	0.64	2.54* (2.36, 2.71)	2.51 (2.26, 2.78)	0.29
Morning break	45.43 ^¥^* (43.24, 47.62)	62.32 (58.13, 66.51)	< 0.01	33.78 ^¥^* (30.28, 42.90)	23.65 (17.40, 29.91)	< 0.01	3.12 ^¥^* (2.91, 3.33)	2.74 (2.36, 3.11)	0.04
Late-morning	74.04 ^¥^ (72.50, 75.58)	63.57 (60.49, 66.65)	< 0.01	26.14 (24.81, 27.46)	25.92 (23.69, 28.15)	0.82	2.94* (2.75, 3.12)	3.22 (2.94, 3.51)	0.13
Lunch	48.75 ^¥^* (46.41, 51.08)	61.80 (58.34, 65.26)	< 0.01	24.65* (22.54, 26.77)	25.28 (22.00, 28.55)	0.95	3.20 (3.01, 3.39)	3.25 (2.96, 3.55)	0.74
Early afternoon	73.18 ^¥^* (71.34, 74.83)	64.38 (61.20, 67.56)	< 0.01	26.73* (25.39, 28.08)	25.93 (22.50, 28.96)	0.54	3.16* (2.96, 3.36)	3.32 (3.06, 3.58)	0.64
Late afternoon	62.89 ^¥^ (61.25, 64.53)	66.15 (63.54, 68.75)	< 0.01	23.85 ^¥^ (22.79, 24.91)	25.71 (23.96, 27.47)	0.049	3.33 (3.17, 3.48)	3.27 (3.02, 3.52)	0.29
Evening	72.58 (70.81, 74.35)	73.92 (71.71, 76.14)	0.41	28.93 (27.79, 30.06)	28.04 (26.33, 29.74)	0.21	2.91 (2.77, 3.06)	3.19 (2.98, 3.40)	0.09
Class time	74.61* (73.34, 75.90)	N/A	N/A	25.01 (24.05, 25.97)	N/A	N/A	2.87* (2.71, 3.04)	N/A	N/A
School time	69.5* (68.25, 70.79)	N/A	N/A	25.34* (24.46, 26.22)	N/A	N/A	2.94* (2.79, 3.09)	N/A	N/A
Out-of-school time	64.94 (63.59, 66.30)	N/A	N/A	21.96 (21.27, 22.66)	N/A	N/A	2.49* (2.39, 2.59)	N/A	N/A

**Fig. 2:**
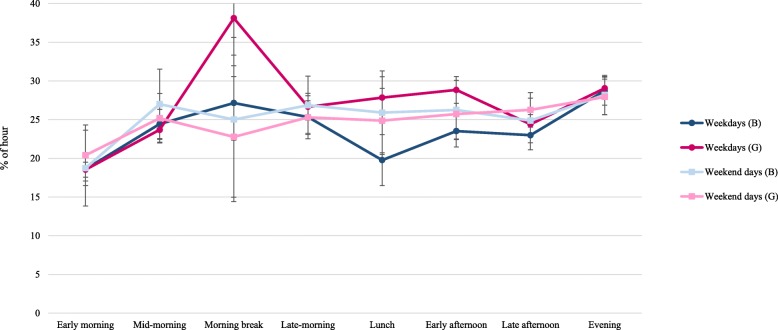
The percentage of each hour within a period spent in prolonged sitting among boys and girls on weekdays and weekend days. Note: B = boys; G = girls. This study was conducted in Melbourne, Australia, between August 2014–December 2015

**Fig. 3:**
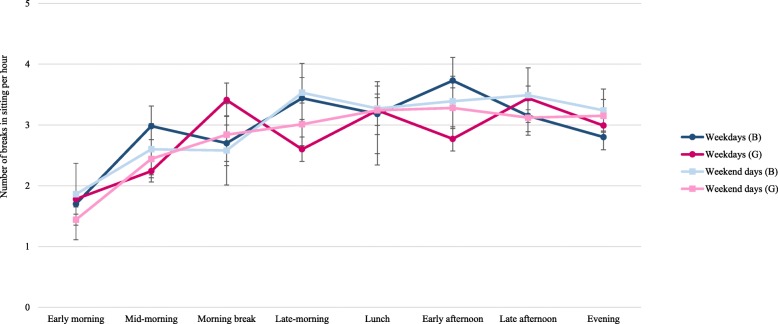
The number of sitting breaks per hour within a period for boys and girls on weekdays and weekend days. Note: B = boys; G = girls. This study was conducted in Melbourne, Australia, between August 2014–December 2015

Differences between weekdays and weekend days examined by t-test, ^¥^indicates significant differences between day type. (*) indicates significant sex differences, see Figs. 1, 2, 3 and Additional file 1: Table S1, Additional file 2: Table S2, Additional file 3: Table S3. ^a^Includes only those with 8 h of wear time on at least 3 weekdays; ^b^Includes only those with 7 h of wear time on at least one weekend day. This study was conducted in Melbourne, Australia, between August 2014–December 2015

### Prolonged sitting

The percentage of time (per hour) in prolonged sitting (i.e. a sitting bout lasting ≥10 min) within each period ranged from 18.6 to 33.8% on weekdays and 19.7 to 28% on weekend days (Table 2). On both weekdays and weekend days, participants performed the least amounts of prolonged sitting in the early morning period and the greatest amount in the evening period. There were significant differences in prolonged sitting on weekdays and weekend days during the morning break (33.8% vs 23.7%, respectively) and late afternoon (23.9% vs 25.7%, respectively). There were also sex differences in prolonged sitting in four periods on weekdays with girls performing more than boys during the school time (26.7% vs 23.3%), morning break (38.1% vs 27%), lunch (27.9% vs 19.8%) and early afternoon (28.8% vs 23.5%) (Fig. 2 and Additional file 2: Table S2). There were no sex differences in prolonged sitting during any period on weekend days.

### Sitting breaks

Across periods, participants performed 1.8–3.3 breaks in sitting per hour on weekdays and 1.6–3.3 breaks in sitting per hour on weekend days (Table 2). There were significantly more breaks in sitting during the morning break period on weekdays (3.12/h) compared to weekend days (2.74/h). There were sex differences in the number of sitting breaks per hour for eight weekday periods (Fig. 3 and Additional file 3: Table S3) with girls performing more sitting breaks per hour during the early morning (1.8 vs 1.7), out-of-school time (2.6 vs 2.4) and morning break (3.4 vs 2.7) periods, whereas boys performed more sitting breaks during class time (3.4 vs 2.5), school time (3.3 vs 2.7), mid-morning (3 vs 2.2), late-morning (3.4 vs 2.6) and early afternoon (3.7 vs 2.8) periods. There were no sex differences in sitting breaks per hour across any period on weekend days.

## Discussion

This study provides a novel analysis of activPAL-measured patterns of sitting (bouts and breaks) among adolescents. Findings show that the majority of adolescents’ waking time is spent sitting and much of this occurs in prolonged bouts. The structure of the school day appears to influence sitting patterns with distinct differences on weekdays and weekend days, and by sex. Class time, school break times, and the evening period (6 pm–10 pm) are identified as key periods requiring strategies to reduce adolescent’s sitting. Interestingly, despite the structure the school timetable provides, there are differences between boys and girls during class time which may necessitate sex-specific intervention strategies.

Sitting patterns identified in this study were generally consistent with other *activ*PAL™ studies in adolescents. Overall, adolescents in the current study performed similar amounts of daily *activ*PAL™ measured sitting (68%) to a small sample of Scottish adolescents (65%) [17] and female Irish adolescents (65%) [18, 24]. Adolescents in this study spent a similar proportion of the day sitting on week- and weekend days, consistent with findings by Hughes and colleagues [17], and almost a third of their time in prolonged sitting, in line with 10–16 year-olds in the US who spend 29% of their time in accelerometer- measured sedentary bouts [13]. To the authors’ knowledge, only one other study has used *activ*PALs™ to examine the number of breaks in sitting among adolescents. In that study, participants performed 54 breaks/day [17]. Similarly, adolescents in the current study would perform approximately 50 breaks in sitting per day if the findings were extrapolated to 16 h of waking time. The current study builds upon these findings by identifying when the sitting breaks are occurring (e.g. adolescents performed 1.8 breaks per hour in the early morning period which may be when they are moving between classes, and 3.3 breaks per hour in the late afternoon period when they are no longer restricted by the school timetable). This information would be useful for intervention development as it highlights the periods when strategies to promote breaking up sitting may be most needed. It also highlighting when adolescents are currently able to break- up sitting and therefore, where maintenance strategies may be considered.

The day of the week (i.e. weekday or weekend) and the time of day (class time or break times) are important influences on adolescents’ sitting time and patterns as there is less variation during periods on weekend days (e.g. sitting for 62–74% of a period), but more variability during periods on weekdays (sitting for 45–75% of a period). This suggests that the school timetable is an important contributor to adolescents’ sitting patterns, particularly for girls. Given boys and girls are exposed to the same school timetable, the sex differences in class time sitting are intriguing. It appears that once in class, girls remain seated for longer with fewer breaks in sitting and a greater amount of time spent in prolonged sitting than boys. In comparison, boys may find a way to move during class (e.g. fidget and stand) or teachers may deliberately find ways for the boys to move. A greater understanding of why these differences exist is needed. Interventions targeting school-based sitting (e.g. standing/active classes) amongst children have shown promise [33], however there is less evidence for their effectiveness amongst adolescents [34, 35]. The findings from the current study highlight the need for interventions to target school based sitting among this population group.

Although both boys and girls accumulated less overall and less prolonged sitting per hour during the morning break and lunchtimes, when there are more opportunities to be active compared to class time, girls still performed significantly more sitting than boys. Many reviews have identified consistent differences in physical activity among boys and girls, with boys nearly always more active [36–38]. Few studies, however, have reported sex differences in objectively assessed sitting and sitting patterns (i.e., bouts and breaks). The current findings highlight the need for interventions to target girls’ sitting during school breaks. For example, interventions targeting female adolescents’ behaviours during the short morning break (i.e. 20–30 min) such as the WISH peer-lead walking at recess program, have been shown to successfully reduce sedentary behaviours by (9 min/school day) [39], and may have a large impact on adolescent girls’ sitting across an entire week.

The evening (6 pm–10 pm) emerged as another key period of the day, when adolescents have the greatest out-of-school hours sitting and prolonged sitting. During this period adolescents may have opportunities to engage in both screen (e.g. TV’s, digital tablets, mobile phones) and non-screen based sitting behaviours (e.g. writing homework, hanging out with friends) for leisure and homework purposes [40]. Intervening during this period may be challenging due to varying home environments, dinner-time schedules, reducing daylight and adolescents’ homework requirements. However, considered intervention strategies that, for example, encourage adolescents to reduce sitting during daylight hours (e.g. a walk before/after dinner) or encourage regular breaks from sitting while completing homework or watching TV, may improve adolescent’s sitting patterns during this period.

The strengths of this study include the objective measurement of sitting, examination of sitting patterns during different day periods, and the large sample of objectively measured sitting amongst boys and girls. Further, examining the percentage of each period sitting, instead of raw minutes, and the standardising of sitting breaks and prolonged sitting to per-hour within a period enables greater comparisons to other studies. Generalisability of the findings to other countries may be challenging, however, because of differences in the structure of the school day. Participants were required to remove the *activ*PAL™ for water-based activities which is a limitation. However, they would be unlikely to be sitting during these activities so it should have had minimal impact on sitting estimates. Investigation into the types of sedentary behaviours boys and girls perform while sitting (e.g. reading, using a digital tablet, sitting and talking), during sitting breaks (e.g. standing still, light-intensity movement) and the additional contextual information relating to these behaviours (e.g. who with, where within the school/home are they located) would be useful for informing intervention development. Further, the start and finish times of the day (6 am-10 pm) would not capture behaviours performed outside of these hours and a 24 h wearing protocol may be preferable in future studies. Since the current data collection, it has also been suggested that a longer wear time period (9-days) be required for the measurement of adolescents’ sitting [41]. Future research should also examine longitudinal changes in adolescents’ sitting patterns, identify how adolescents’ sitting patterns affect their health, and test intervention strategies targeting these suboptimal behaviour patterns throughout the day.

## Conclusion

This study provided a novel exploration of objectively- measured patterns of sitting among adolescents, finding that irrespective of sex, adolescents spent the majority of their time sitting, particularly in prolonged sitting. Differences in sitting patterns between boys and girls, weekdays and weekend days, between class times, school breaks and the evening were found. Key periods identified for intervention include class time, school breaks (particularly for girls) and the evening period where strategies are needed to reduce adolescent’s sitting.

## Additional files

Additional file 1: **Table S1:** Differences in the percentage of the period (mean, 95%CI) spent sitting in each period by sex and weekdays or weekend days. This table shows the sex and day (week day or weekend day) differences in the percentage of each period (mean, 95%CI) spent sitting. (DOCX 14 kb)

Additional file 2: **Table S2:** Differences in the percentage of time (per hour) spent sitting in a bout of ≥10 min (mean, 95%CI) by sex and weekdays or weekend days. This table shows the sex and day (week day or weekend day) differences in the time (per hour) spent sitting in a bout of ≥10 min (mean, 95%CI). (DOCX 14 kb)

Additional file 3: **Table S3:** Differences in frequency of sitting breaks per hour in each period (mean, 95%CI) by sex and weekdays or weekend days. This table shows the sex and day (week day or weekend day) differences in the frequency of sitting breaks per hour in each period (mean, 95%CI). (DOCX 14 kb)

## Abbreviations

HEAG-H: The Deakin University Human Ethics Advisory Group
NEArbY: The NEighbourhood Activity in Youth Project
SA1: Level 1 Statistical Areas
