# Supplementary material for: Exploring when and how adolescents sit: cross-sectional analysis of activPAL-measured patterns of daily sitting time, bouts and breaks
Source: BMC Public Health. 2019 Jun 11;19:653. doi: 10.1186/s12889-019-6960-5 (PMC6558889; doi:10.1186/s12889-019-6960-5)
Supplement: Supplementary file 1 — Table S1: Differences in the percentage of the period (mean, 95%CI) spent sitting in each period by sex and weekdays or weekend days. This table shows the sex and day (week day or weekend day) differences in the percentage of each period (mean, 95%CI) spent sitting. (DOCX 14 kb) [file 12889_2019_6960_MOESM1_ESM.docx]

**Additional Table 1:** Comparisons of the percentage of the period (mean, 95%CI) spent sitting in each period by sex and weekdays or weekend days

|  | Weekdays | | | Weekend days | | |
| --- | --- | --- | --- | --- | --- | --- |
|  | Boys | Girls | p for sex | Boys | Girls | p for sex |
| Early morning | 64.05 ^¥^  (61.15, 66.96) | 63.31  (61.26, 65.37) | 0.68 | 71.96  (64.62, 79.30) | 69.32  (62.11, 76.53) | 0.61 |
| Mid-morning | 73.32  (70.55, 76.09) | 75.42 ^¥ ¥^  (73.42, 77.42) | 0.21 | 70.03  (64.82, 75.25) | 64.24  (59.31, 69.18) | 0.12 |
| Morning break | 41.36 ^¥ ¥*^  (37.90, 44.81) | 48.19 ^¥ ¥^  (45.41, 50.98) | <0.01 | 67.16  (60.76, 73.56) | 58.99  (53.46, 64.52) | 0.06 |
| Late-morning | 73.20 ^¥^  (70.55, 75.84) | 74.61 ^¥ ¥^  (72.73, 76.48) | 0.38 | 65.96  (61.38, 70.53) | 61.95  (57.78, 66.11) | 0.21 |
| Lunch | 43.70 ^¥ ¥*^  (39.83, 47.57) | 52.17 ^¥ ¥^  (49.32, 55.01) | <0.01 | 63.84  (58.50, 69.18) | 60.42  (55.85, 64.98) | 0.34 |
| Early afternoon | 69.23*  (66.26, 72.20) | 75.87 ^¥ ¥^  (74.05, 77.68) | <0.01 | 67.61  (63.94, 72.28) | 62.19  (57.89, 66.50) | 0.10 |
| Late afternoon | 62.43  (59.75, 65.11) | 63.19 ^¥^  (61.11, 65.28) | 0.65 | 64.17  (60.31, 68.03) | 67.49  (63.97, 71.00) | 0.22 |
| Evening | 73.58 ^¥^  (70.87, 76.30) | 71.93  (69.58, 74.27) | 0.37 | 73.95  (70.47, 77.43) | 73.91  (71.00, 76.82) | 0.99 |
| Class time | 72.42*  (70.14, 74.71) | 76.11  (74.64, 77.58) | <0.01 | N/A | N/A | N/A |
| School time | 66.58*  (64.32, 68.8) | 71.53  (70.10, 72.96) | <0.01 | N/A | N/A | N/A |
| Out-of-school time | 64.32  (62.07, 66.57) | 65.35  (63.65, 67.06) | 0.47 | N/A | N/A | N/A |

Significant differences between weekday and weekday indicated by ^¥^ ^¥^ = p<0.01, ^¥^ = p<0.05, (*) indicates significant sex differences within week or weekend days. This study was conducted in Melbourne, Australia, between August 2014 - December 2015. The average length of the periods were: *early morning:* 2hrs, 52mins; *mid-morning:* 1hr, 51mins; *morning break*: 25mins; *late morning*: 1hr, 41mins), *lunch*: 47mins; *early afternoon*: 1hr, 34mins; *late afternoon*: 2hrs, 53mins; *evening*: 4hrs; *weekday class time* (sum of mid-morning, late morning and early afternoon): 5hr, 6mins; *school time* (sum of mid-morning, morning break, late morning, lunch and early afternoon): 6hrs, 18mins); and *outside of school time* (sum of early morning, late afternoon and evening): 9hrs, 45mins.
