# Supplementary material for: Exploring when and how adolescents sit: cross-sectional analysis of activPAL-measured patterns of daily sitting time, bouts and breaks
Source: BMC Public Health. 2019 Jun 11;19:653. doi: 10.1186/s12889-019-6960-5 (PMC6558889; doi:10.1186/s12889-019-6960-5)
Supplement: Supplementary file 2 — Table S2: Differences in the percentage of time (per hour) spent sitting in a bout of ≥10 min (mean, 95%CI) by sex and weekdays or weekend days. This table shows the sex and day (week day or weekend day) differences in the time (per hour) spent sitting in a bout of ≥10 min (mean, 95%CI). (DOCX 14 kb) [file 12889_2019_6960_MOESM2_ESM.docx]

**Additional Table 2:** Comparisons of the percentage of time (per hour) spent sitting in a bout of ≥10minutes (mean,95%CI) by sex and week or weekend days

|  | Weekdays | | | Weekend days | | |
| --- | --- | --- | --- | --- | --- | --- |
|  | Boys | Girls | p for sex | Boys | Girls | p for sex |
| Early morning | 18.59  (17.06, 20.11) | 18.53  (17.55, 19.51) | 0.96 | 18.74  (13.84, 23.64) | 20.40  (16.49, 24.30) | 0.59 |
| Mid-morning | 24.44  (22.54, 26.33) | 23.68  (22.09, 25.27) | 0.55 | 27.00  (22.46, 31.54) | 25.18  (21.98, 28.38) | 0.51 |
| Morning break | 27.15*  (22.34, 31.95) | 38.11^¥¥^  (33.33, 42.90) | <0.01 | 25.01  (14.41, 35.60) | 22.77  (14.97, 30.57) | 0.73 |
| Late-morning | 25.33  (23.24, 27.43) | 26.66  (24.94, 28.39) | 0.34 | 26.85  (23.09, 30.62) | 25.30  (22.52, 28.09) | 0.50 |
| Lunch | 19.77*  (16.48, 23.07) | 27.85  (25.15, 30.54) | <0.01 | 25.90  (20.50, 31.29) | 24.87  (20.70, 29.03) | 0.76 |
| Early afternoon | 23.52*  (21.47, 25.57) | 28.84  (27.11, 30.57) | <0.01 | 2*6.25  (22.41, 30.08) | 25.73  (22.50, 28.96) | 0.84 |
| Late afternoon | 22.99  (21.11, 24.87) | 24.40  (23.15, 25.66) | 0.20 | 24.88  (22.01, 27.77) | 26.26  (24.03, 28.49) | 0.45 |
| Evening | 28.72  (26.87, 30.58) | 29.05  (27.61, 30.50) | 0.78 | 28.19  (25.65, 30.74) | 27.93  (25.63, 30.24) | 0.88 |
| Class time | 23.87  (22.38, 25.36) | 25.77  (24.51, 27.02) | 0.06 | N/A | N/A | N/A |
| School time | 23.35*  (22.01, 24.68) | 26.66  (25.52, 27.79) | <0.01 | N/A | N/A | N/A |
| Out-of-school time | 21.10  (19.81, 22.39) | 22.51  (21.72, 23.30) | 0.07 | N/A | N/A | N/A |

Significant differences between weekday and weekday indicated by ^¥¥^= p<0.01, ^¥^= p<0.05; (*) indicates significant sex differences within week or weekend days. This study was conducted in Melbourne, Australia, between August 2014 - December 2015. The average length of the periods were: *early morning:* 2hrs, 52mins; *mid-morning:* 1hr, 51mins; *morning break*: 25mins; *late morning*: 1hr, 41mins), *lunch*: 47mins; *early afternoon*: 1hr, 34mins; *late afternoon*: 2hrs, 53mins; *evening*: 4hrs; *weekday class time* (sum of mid-morning, late morning and early afternoon): 5hr, 6mins; *school time* (sum of mid-morning, morning break, late morning, lunch and early afternoon): 6hrs, 18mins); and *outside of school time* (sum of early morning, late afternoon and evening): 9hrs, 45mins.
